# Kappa free light chain index in multiple sclerosis and other inflammatory CNS diseases: a pilot single-center study from Argentina

**DOI:** 10.3389/fimmu.2026.1803446

**Published:** 2026-05-29

**Authors:** Analisa Manin, Fernanda Ingenito, Evangelina Valeria Cores, Diego Alarcón Guerrero, Silvia Graciela Ramos, Andrés M. Villa

**Affiliations:** 1 Neuroimmunology Section, Neurology Division, J. M. Ramos Mejía Hospital; University of Buenos Aires (UBA), Buenos Aires, Argentina; 2 Argentine Center for Neuroimmunological Diseases (CADENI), Faculty of Medicine, University of Buenos Aires (UBA), Buenos Aires, Argentina; 3 Immunology Section, Neuroimmunology Area, Carlos G. Durand General Acute Care Hospital, Buenos Aires, Argentina; 4 Research Institute, Faculty of Medicine, University of Buenos Aires, Buenos Aires, Argentina

**Keywords:** cerebrospinal fluid, IgG index, multiple sclerosis, oligoclonal bands, κ-FLC index, kappa free light chain, intrathecal immunoglobulin synthesis

## Abstract

**Background:**

Intrathecal immunoglobulin synthesis is a hallmark of multiple sclerosis (MS) and can be assessed using the kappa free light chain (κ-FLC) index. Although widely adopted, the optimal diagnostic cut-off of the κ-FLC index may vary according to the clinical context and comparison group.

**Objective:**

To evaluate the diagnostic performance of the κ-FLC index for MS in a real-world series including inflammatory and non-inflammatory neurological disorders.

**Methods:**

We conducted a retrospective cross-sectional study including consecutive patients aged ≥16 years undergoing diagnostic lumbar puncture at a tertiary public hospital in Argentina. Patients were classified as MS/clinically isolated syndrome (MS/CIS), other inflammatory neurological diseases (OIND), or non-inflammatory neurological diseases (NIND). κ-FLC, IgG, and albumin were measured in paired cerebrospinal fluid and serum samples, and κ-FLC index was calculated using quotient-based normalization. In addition, intrathecal κ-FLC synthesis was assessed using the hyperbolic Reiber diagram (Reiber’s diagram). Diagnostic performance was assessed using receiver operating characteristic (ROC) curves, with cut-off values derived from Youden’s index and compared with previously proposed thresholds.

**Results:**

A total of 124 patients were included (49 MS/CIS, 48 OIND, 27 NIND). Median κ-FLC index values were significantly higher in MS/CIS compared with OIND and NIND (p < 0.001). In the primary comparison between MS/CIS and OIND, the optimal κ-FLC index cut-off was 14.6, yielding a sensitivity of 73.5% and a specificity of 68.1%. The lower threshold of 6.1 showed higher sensitivity (85.7%) but limited specificity (35.4%), while Reiber’s diagram for κ-FLC showed the highest sensitivity (91.8%) but lower specificity (45.8%).

**Conclusions:**

The κ-FLC index is a valuable quantitative biomarker of intrathecal immunoglobulin synthesis in MS. In our setting, a higher κ-FLC index threshold provided a more balanced diagnostic profile, whereas Reiber’s diagram favored sensitivity. Higher κ-FLC index thresholds may improve diagnostic specificity in real-world clinical settings and should be validated in independent series.

## Introduction

Multiple sclerosis (MS) is a chronic immune-mediated disease of the central nervous system (CNS) characterized by inflammatory demyelination and neuroaxonal injury. Diagnosis relies on demonstrating dissemination in time and space using clinical features and MRI, with cerebrospinal fluid (CSF) biomarkers providing supportive evidence of intrathecal immune activation (1, 2).

Intrathecal immunoglobulin synthesis occurs across several inflammatory CNS disorders and has well-established diagnostic value in MS (1). Kappa free light chains (κ-FLC) are produced in excess during immunoglobulin assembly and are released as free molecules, making them a practical surrogate of intrathecal humoral immune activity (2–4). Measurement of κ-FLC in paired CSF and serum samples, typically expressed as an index adjusted for blood–CSF barrier function, helps distinguish intrathecal synthesis from passive diffusion from the peripheral circulation.

Traditionally, intrathecal immunoglobulin synthesis has been assessed by oligoclonal IgG bands (OCBs) and the immunoglobulin G (IgG) index (5–7). Despite their widespread use, these approaches are qualitative or semi-quantitative, may show incomplete inter-laboratory standardization, and often require expert interpretation (7).

In recent years, the κ-FLC index has emerged as a promising quantitative biomarker for intrathecal immunoglobulin synthesis (1–3, 8). Several studies have reported diagnostic performance comparable to OCBs in MS, with additional advantages including automation, technical simplicity, and reproducibility (8–10).

Hyperbolic quotient diagrams based on the Reiber model have also been proposed as an alternative method to define intrathecal k-FLC synthesis, aiming to better distinguish true intrathecal production from passive transfer across the blood–CSF barrier (11, 12). Recent evidence has also highlighted that OCBs and κ-FLC should not necessarily be viewed as competing biomarkers, but rather as complementary tools for assessing intrathecal humoral immune activity. In this framework, OCBs retain the advantage of long-standing clinical validation and pattern-based interpretation, whereas κ-FLC offers a quantitative, rapid, and potentially more scalable alternative whose diagnostic performance is influenced by the selected analytical approach and cut-off definition (12).

However, elevated intrathecal κ-FLC synthesis is not specific to MS and can also be observed in other inflammatory neurological diseases (OIND), including neuromyelitis optica spectrum disorder, myelin oligodendrocyte glycoprotein antibody–associated disease, and autoimmune encephalitis (13–16). Because κ-FLC testing is increasingly used in routine practice for patients with suspected inflammatory CNS disease, defining clinically meaningful, population- and assay-adapted cut-off values is essential—particularly when differentiating MS/CIS from both non-inflammatory neurological disorders and other inflammatory conditions.

The present study aimed to assess the diagnostic performance of the κ-FLC index in a single-center Argentinian population including patients with MS/clinically isolated syndrome, other inflammatory neurological diseases, and non-inflammatory neurological disorders, and to derive clinically meaningful cut-off values for our setting.

## Materials and methods

### Study design and setting

The presence of oligoclonal bands (OCBs), serum and cerebrospinal fluid (CSF) albumin, serum and CSF immunoglobulin G (IgG), serum and CSF kappa free light chains (κ-FLCs) were recorded for all consecutive patients who underwent diagnostic lumbar puncture with assessment of intrathecal immunoglobulin synthesis as part of the evaluation of suspected central nervous system (CNS) disorders. Patients evaluated at the Neuroimmunology Section of Hospital J. M. Ramos Mejía in Buenos Aires city between August 2023 and August 2025 were retrospectively included.

### Participants

Eligible participants were aged ≥16 years, presented with a first clinical episode suggestive of an inflammatory neurological disorder, and were admitted to the Neurology ward for diagnostic evaluation and treatment. All patients underwent diagnostic lumbar puncture as part of their routine clinical work-up.

Paired blood and cerebrospinal fluid (CSF) samples were obtained prior to the initiation of any immunosuppressive or immunomodulatory therapy, including both acute and chronic treatments. An aliquot of serum and CSF samples collected for routine clinical purposes was retained for biomarker analysis.

Exclusion criteria included pregnancy, prior exposure to acute or chronic immunosuppressive or immunomodulatory treatment, including corticosteroids, plasma exchange, or intravenous immunoglobulins, and conditions that could interfere with κ-FLC concentrations, such as blood contamination, monoclonal gammopathies, and impaired renal function (17–19).

### Diagnostic classification

Participants were classified into three groups based on the final clinical diagnosis. Multiple sclerosis/clinically isolated syndrome (MS/CIS). This group included patients fulfilling the 2017 McDonald diagnostic criteria for multiple sclerosis, including those presenting with a clinically isolated syndrome (20). Other inflammatory neurological diseases (OIND). The non-MS inflammatory group comprised patients with other inflammatory neurological diseases diagnosed according to established international criteria. This group included patients with neuromyelitis optica spectrum disorder (NMOSD) and myelin oligodendrocyte glycoprotein antibody–associated disease (MOGAD), diagnosed according to the 2015 International Panel criteria for NMOSD (21) and the 2023 international consensus criteria for MOGAD (22), respectively, as well as other immune-mediated inflammatory neurological conditions (23). Non-inflammatory neurological diseases (NIND). This group included patients in whom inflammatory, autoimmune, infectious, and demyelinating etiologies were excluded after comprehensive clinical, radiological, and laboratory evaluation. Diagnoses in this group comprised non-inflammatory headache disorders, nonspecific white matter changes, degenerative neurological conditions, compressive myelopathies, and other non-inflammatory neurological disorders.

Final diagnostic classification was performed by experienced neuroimmunologists based on all available clinical, neuroimaging, and laboratory data. Details regarding diagnostic composition and group sizes are provided in the Results section.

Antibody testing for aquaporin-4 (AQP4) and myelin oligodendrocyte glycoprotein (MOG) was performed when clinically indicated using commercially available fixed cell-based assays.

Demographic and clinical variables collected from medical records included age at sampling, sex, Expanded Disability Status Scale (EDSS), and final diagnostic classification.

The study protocol was approved by the Institutional Review Board (IRB) of Hospital J. M. Ramos Mejía.

### Sample collection and laboratory analysis

CSF and serum samples were obtained during initial diagnostic work-up or for diagnostic confirmation. Samples were centrifuged at 3600 rpm for 6 minutes and stored at 2 °C–8 °C for ≦̸ 2 days or frozen at −80 °C.

All laboratory analyses were performed at the Immunology Laboratory of Hospital General de Agudos Carlos G. Durand. Concentrations of kappa free light chains (κ-FLC), immunoglobulin G (IgG), and albumin in paired CSF and serum samples were quantified by immunoturbidimetry using an automated analyzer (SpaPlus^®^, The Binding Site Group Ltd., Birmingham, UK), in accordance with the manufacturer’s instructions.

To account for blood–CSF barrier function and distinguish intrathecal immunoglobulin synthesis from passive diffusion, quotient-based indices were calculated. The albumin quotient (QAlb) was calculated as the ratio of CSF albumin to serum albumin and used as a marker of blood–CSF barrier integrity. The κ-FLC quotient (Qκ-FLC) was calculated as the ratio of CSF κ-FLC to serum κ-FLC concentrations. Intrathecal immunoglobulin synthesis was assessed using the following formulas:

-
IgG index=QIgG/QAlb.
-
κ−FLC index=Qκ−FLC/QAlb.

Where:

-
QIgG=CSF IgG/serum IgG.
-
Qκ−FLC=CSF κ−FLC/serum κ−FLC.
-
QAlb=CSF albumin/serum albumin.

In addition, Reiber’s diagram was assessed using the hyperbolic reference range described by Reiber et al. for κ-FLC in CSF/serum quotient diagrams. The upper reference limit for the κ-FLC quotient was calculated as Qκ(lim) = [3.27 × √((QAlb × 1000)^2 + 33) − 8.2]/1000. Intrathecal κ-FLC synthesis by Reiber was considered present when Qκ exceeded Qκ(lim).

Oligoclonal IgG bands (OCBs) were detected by isoelectric focusing on agarose gel using a semi-automated Hydrasys system (SEBIA), followed by immunofixation with peroxidase-labeled anti-IgG antiserum. OCB positivity was defined as the presence of ≥2 CSF-restricted bands. All laboratory results were independently reviewed by two experienced laboratory specialists.

### Statistical analysis

Quantitative variables were summarized as median and interquartile range (IQR), while categorical variables were expressed as absolute frequencies and percentages. Comparisons of continuous variables across diagnostic groups (MS/CIS, OIND, and NIND) were performed using the Kruskal–Wallis test. When overall group differences were identified, *post hoc* pairwise comparisons were conducted using the Bonferroni correction to adjust for multiple testing. Categorical variables were compared using the χ² test or Fisher’s exact test, as appropriate.

Receiver operating characteristic (ROC) curves were generated for each biomarker, and the area under the curve (AUC) was calculated using IBM SPSS Statistics version 25.0 (IBM Corp., Armonk, NY, USA). The optimal κ-FLC index cut-off was determined using Youden’s index (sensitivity + specificity − 1). In addition to the optimal cut-off derived from the present dataset, a κ-FLC index threshold of 6.1—previously proposed in the literature as maximizing sensitivity for MS diagnosis—was also evaluated. Reiber’s diagram for κ-FLC was analyzed as a binary variable based on the presence of intrathecal κ-FLC synthesis in the quotient diagram. OCB status was analyzed as a binary categorical variable, with positivity defined by the presence of CSF-restricted oligoclonal bands (pattern II or III). For the IgG index, a cut-off value of 0.7 was used in accordance with prior studies.

Multivariable logistic regression models were constructed to evaluate the association between the κ-FLC index (modeled as a continuous variable) and MS/CIS diagnosis, adjusting for age and sex. For OCB positivity, the selected κ-FLC index thresholds, IgG index, and Reiber’s diagram for κ-FLC, sensitivity, specificity, positive predictive value (PPV), negative predictive value (NPV), and likelihood ratios were calculated using contingency tables, with MS/CIS serving as the reference diagnosis.

*A priori* sample size calculation was performed using G*Power software to ensure adequate statistical power to detect significant differences between groups, particularly between MS and OIND. Based on a recently published study with a comparable methodology, an estimated odds ratio of 9.5, a significance level (α) of 0.05, and a target statistical power (1−β) of 0.99 were assumed. This calculation indicated a minimum required sample size of 34 participants (17 per group). To avoid limiting the analysis to the minimum statistical requirement and to enhance the representativeness of the findings, consecutive recruitment was extended to include all eligible patients evaluated during the predefined recruitment period. All statistical tests were two-tailed, and statistical significance was set at p < 0.05.

## Results

A total of 124 patients were included in the study. Of these, 49 were diagnosed with MS/CIS, 48 were classified as having other inflammatory neurological diseases (OIND), and 27 as having non-inflammatory neurological diseases (NIND). Demographic and biomarker characteristics are summarized in **Table 1**. Median age differed significantly among groups, being lowest in MS/CIS (34.2 years; IQR 28.1–43.1), intermediate in OIND (43.0 years; IQR 29.4–49.9), and highest in NIND (47.5 years; IQR 30.9–56.2) (p < 0.001). A female predominance was observed across all groups, with no significant differences in sex distribution (p = 0.53).

**Table 1:** Baseline characteristics of the study population.

Group	n	Age, years, median (IQR)	Female sex, n (%)	EDSS, median (IQR)
**MS/CIS**	49	34.2 (28.1–43.1)	33 (67.3)	1.25 (0.0–3.0)
**OIND total**	48	43.0 (29.4–49.9)	36 (75.0)	
NMOSD	17	42.5 (29–50)	15 (88.2)	3 (1.5–3.5)
MOGAD	7	43 (20–45)	5 (71.4)	1.5 (0–2)
Autoimmune OICDs	18	40.5 (20–45)	13 (72.2)	—
Infectious OICDs	6	46 (32.5–52.1)	3 (50.0)	—
**NIND**	27	47.5 (30.9–56.2)	17 (63.0)	—

CIS, clinically isolated syndrome; EDSS, Expanded Disability Status Scale; IQR, interquartile range; MOGAD, myelin oligodendrocyte glycoprotein antibody–associated disease; MS, multiple sclerosis; NIND, non-inflammatory neurological diseases; OICDs, other inflammatory central nervous system diseases; OIND, other inflammatory neurological diseases; NMOSD, neuromyelitis optica spectrum disorder. P values: age, p <0.001; female sex, p =0.53. Bold terms indicate the main diagnostic groups; non-bold rows indicate subgroups within each category.

**Figure 1:**
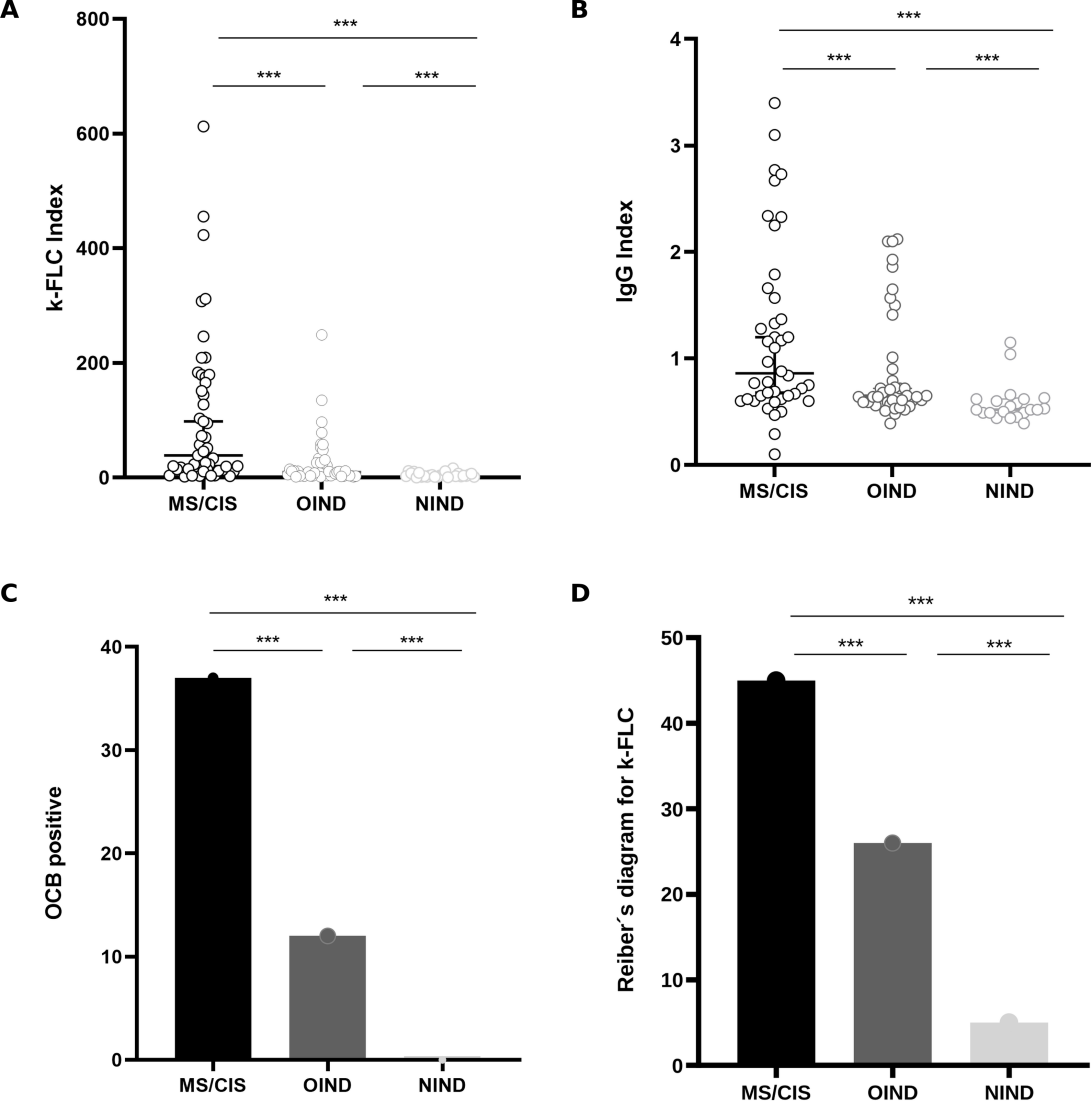
Distribution of CSF biomarkers across diagnostic groups. **(A)** K-FLC index values were highest in MS/CIS (median 41.96; IQR 12.63-165.53), intermediate in OIND (8.75; IQR 3.16-25.84), and lowest in NIND (3.36; IQR 2.43-6.76) (p < 0.001). **(B)** IgG index showed a similar gradient: MS/CIS (0.86; IQR 0.62-1.17), OIND (0.67; IQR 0.59-1.11), and NIND (0.53; IQR 0.49-0.63) (p < 0.001). **(C)** OCB positivity was most frequent in MS/CIS (75.5%), followed by OIND (25%), and absent in NIND (0%) (p < 0.001). **(D)** Reiber’s diagram for K-FLC was most frequent in MS/CIS (91.8%), followed by OIND (54.2%), and lowest in NIND (18.5%) (p < 0.001). ***p < 0.001.

Differences in intrathecal immunoglobulin synthesis biomarkers were observed among diagnostic categories (**Table 2**). The κ-FLC index showed the highest values in MS/CIS (median 41.96; IQR 12.63–165.53), lower values in OIND (median 8.75; IQR 3.16–25.84), and the lowest values in NIND (median 3.36; IQR 2.43–6.76) (p < 0.001) (**Figure 1A**). A similar but less pronounced pattern was observed for the IgG index, with median values of 0.86 (IQR 0.62–1.17) in MS/CIS, 0.67 (IQR 0.59–1.11) in OIND, and 0.53 (IQR 0.49–0.63) in NIND (p < 0.001) (**Figure 1B**). Oligoclonal bands (OCB) positivity also differed significantly between groups, being detected in 37 of 49 patients with MS/CIS (75.5%), 12 of 48 patients with OIND (25.0%), and in none of the patients with NIND (p < 0.001) (**Figure 1C**). Reiber-defined intrathecal κ-FLC synthesis was detected in 45 of 49 patients with MS/CIS (91.8%), 26 of 48 with OIND (54.2%), and 5 of 27 with NIND (18.5%) (p < 0.001) (**Figure 1D**).

**Table 2:** CSF and serum biomarkers by diagnostic category.

Diagnostic group	CSF albumin (mg/dL)	Serum albumin (mg/dL)	CSF κ-FLC (mg/L)	Serum κ-FLC (mg/L)	QAlb	Qκ	IgG index	κ-FLC index	OCBs positive, n (%)	Reiber’s diagram for κ-FLC, n (%)
**MS/CIS (n=49)**	20.37 (15.90–29.50)	4245.5 (4003.3–4516.0)	0.27 (0.09–1.28)	1.47 (1.18–1.88)	0.0050 (0.0039–0.0071)	0.22 (0.07–0.76)	0.86 (0.62–1.17)	41.96 (12.63–165.53)	37 (75.5)	45 (91.8)
**OIND, total (n=48)**	19.50 (14.00–28.00)	3930.5 (3130.8–4394.5)	0.09 (0.03–0.28)	1.93 (1.01–2.80)	0.0053 (0.0039–0.0073)	0.046 (0.026–0.137)	0.67 (0.59–1.11)	8.75 (3.16–25.84)	12 (25.0)	26 (54.1)
NMOSD (n=17)	15.80 (12.96–28.42)	3909.0 (3135.5–4562.0)	0.07 (0.02–0.35)	1.72 (0.87–2.98)	0.0047 (0.0036–0.0064)	0.056 (0.010–0.158)	0.69 (0.58–0.72)	9.54 (3.12–28.63)	4 (23.5)	10 (58.8)
MOGAD (n=7)	19.65 (15.90–23.25)	3663.0 (3041.5–4087.8)	0.04 (0.03–0.19)	0.99 (0.76–1.31)	0.0054 (0.0042–0.0064)	0.046 (0.025–0.214)	0.72 (0.54–1.25)	10.08 (4.59–31.28)	1 (14.3)	4 (57.1)
Other autoimmune OICDs (n=18)	27.50 (15.20–31.75)	4078.0 (3345.3–4421.8)	0.10 (0.03–0.18)	2.15 (1.47–3.46)	0.0065 (0.0037–0.0080)	0.038 (0.026–0.088)	0.63 (0.58–0.93)	6.83 (2.93–17.29)	5 (27.8)	9 (50.0)
Infectious OICDs (n=6)	19.00 (12.56–21.50)	3975.0 (2582.0–4412.0)	0.78 (0.04–1.63)	2.01 (1.44–3.06)	0.0053 (0.0035–0.0067)	0.275 (0.026–0.706)	1.57 (1.03–2.82)	25.38 (6.89–150.49)	2 (33.3)	3 (50.0)
**NIND (n=27)**	21.00 (15.00–29.50)	4038.0 (3431.0–4244.5)	0.03 (0.03–0.04)	1.74 (1.04–2.35)	0.0053 (0.0041–0.0079)	0.020 (0.016–0.038)	0.53 (0.49–0.63)	3.36 (2.43–6.76)	0 (0)	5 (18.5)

Data are presented as median (Q1–Q3). OIND includes neuromyelitis optica spectrum disorder (NMOSD), myelin oligodendrocyte glycoprotein antibody–associated disease (MOGAD), other autoimmune inflammatory CNS disorders, and infectious CNS disorders. QAlb = CSF albumin/serum albumin ratio; Qκ = CSF κ-FLC/serum κ-FLC ratio. OCBs = cerebrospinal fluid–restricted oligoclonal IgG bands. Bold terms indicate the main diagnostic groups; non-bold rows indicate subgroups within each category.

Diagnostic performance analyses were performed using MS/CIS as the reference diagnosis (**Table 3**). In the primary comparison between MS/CIS and OIND, OCB positivity showed a sensitivity of 75.5% and a specificity of 75.0%, with a positive likelihood ratio (LR+) of 3.02. In the same comparison, a κ-FLC index cut-off of ≥14.6 achieved a sensitivity of 73.5% with a specificity of 68.8% and an LR+ of 2.30, whereas a lower κ-FLC index threshold of ≥6.1 increased sensitivity (85.7%) at the expense of markedly reduced specificity (35.4%). Reiber’s diagram for k-FLC showed the highest sensitivity (91.8%), but lower specificity (45.8%), with an LR+ of 1.70. The IgG index demonstrated lower overall diagnostic performance, with a sensitivity of 61.2% and specificity of 58.3%.

**Table 3:** Comparative diagnostic performance of OCBs, κ-FLC index, Reiber positivity, and IgG index across diagnostic groups.

Comparison	Variable	Sensitivity (%)	Specificity (%)	PPV (%)	NPV (%)	LR+	LR−
MS vs OIND	OCB	75.5	75	75.5	75	3.02	0.33
	k-FLC Index 14.6	73.5	68.8	70.6	71.1	2.30	0.39
	k-FLC Index 6.1	85.7	35.4	57.5	70.8	1.33	0.40
	Reiber’s diagram for k-FLC	91.8	45.8	63.4	84.6	1.70	0.18
	IgG Index	61.2	58.3	59.1	61.0	1.48	0.66
MS vs NIND	OCB	75.5	100	100	62.9	∞	0.24
	k-FLC Index 14.6	73.5	96.3	97.3	66.7	19.90	0.28
	k-FLC Index 6.1	85.7	70.4	84.0	73.1	2.90	0.20
	Reiber’s diagram for k-FLC	91.8	81.5	90.0	84.6	4.96	0.10
	IgG Index	61.2	85.2	89.7	52.9	4.33	0.44
MS vs OIND + NIND	OCB	75.5	84	75.5	84	4.72	0.29
	k-FLC Index 14.6	73.5	78.7	69.2	81.7	3.40	0.34
	k-FLC Index 6.1	85.7	48.0	51.9	83.7	1.65	0.30
	Reiber’s diagram for k-FLC	91.8	58.7	59.2	91.7	2.22	0.14
	IgG Index	61.2	68.0	55.3	72.9	1.89	0.57
OIND vs NIND	OCB	25	100	100	42.9	∞	0.75
	k-FLC Index 14.6	33.3	96.3	93.8	44.8	8.62	0.71
	k-FLC Index 6.1	64.6	70.4	79.5	52.8	2.18	0.50
	Reiber’s diagram for k-FLC	54.2	81.5	83.9	50.0	2.92	0.56
	IgG Index	41.7	85.2	85.7	41.9	2.93	0.68

OCBs, oligoclonal bands; κ-FLC, kappa free light chains; LR, likelihood ratio; NPV, negative predictive value; PPV, positive predictive value.

**Figure 2:**
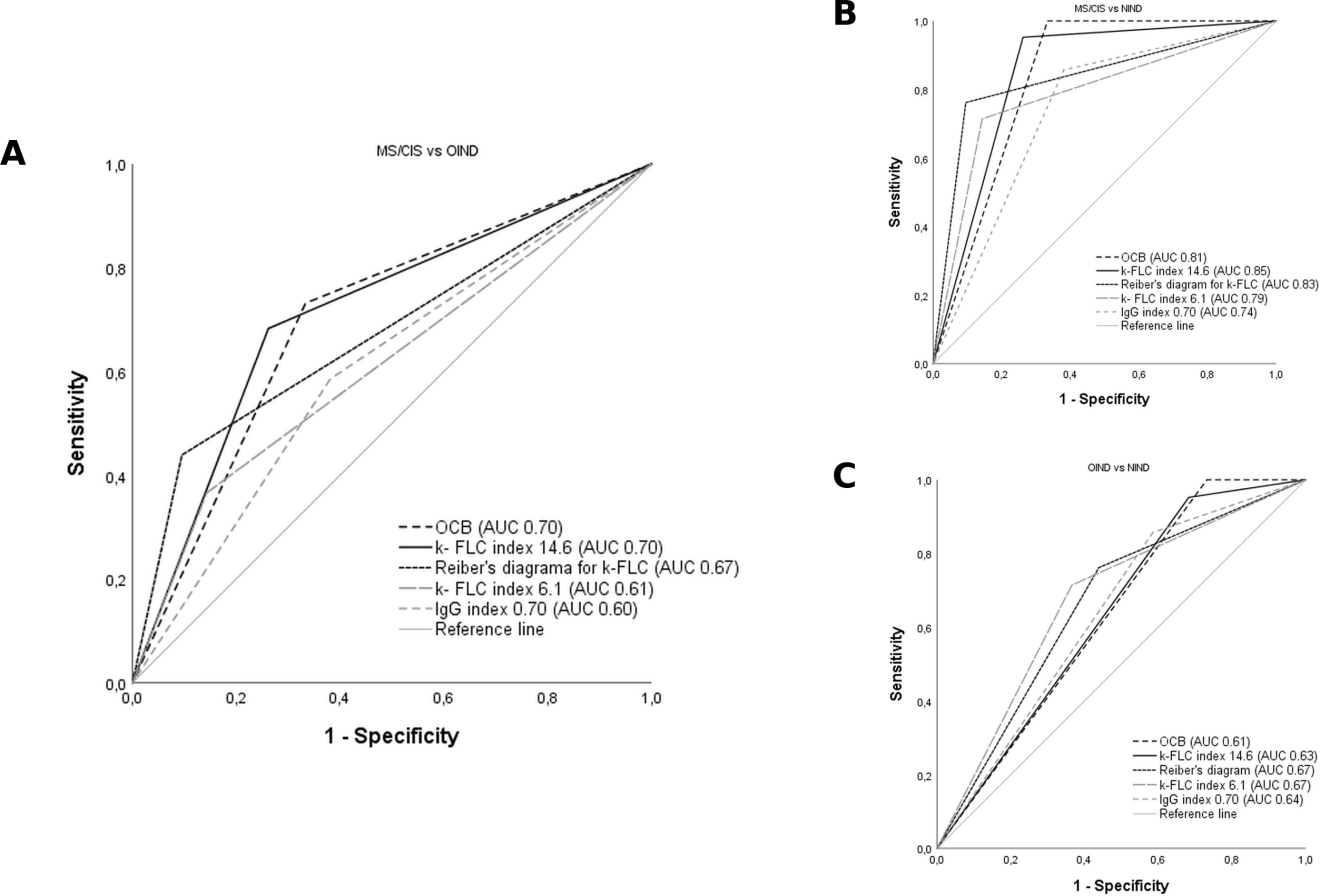
Receiver operating characteristic (ROC) curves comparing the diagnostic performance of cerebrospinal fluid (CSF) biomarkers: K-FLC index (cut-off values ≥6.1 and ≥14.6), Reiber’s diagram for K-FLC, IgG index ≥0.7, and OCBS for differentiating **(A)** multiple sclerosis/clinically isolated syndrome (MS/CIS) vs. other inflammatory neurological diseases (OIND), **(B)** MS/CIS vs. non-inflammatory neurological diseases (NIND), and **(C)** OIND vs. NIND.

Overall discriminative performance in the MS/CIS vs OIND comparison was modest and comparable across markers, with AUCs of 0.711 for κ-FLC index ≥14.6 (95% CI 0.597–0.824; p = 0.001), 0.699 for OCB (95% CI 0.585–0.814; p = 0.002), 0.672 for Reiber’s diagram for κ-FLC (95% CI 0.554–0.789; p = 0.007), 0.611 for κ-FLC index ≥6.1 (95% CI 0.490–0.733; p = 0.080), and 0.602 for IgG index ≥0.70 (95% CI 0.480–0.725; p = 0.109) (**Figure 2**).

When comparing MS/CIS with NIND, OCB positivity showed perfect specificity (100%), with no false-positive results in NIND; accordingly, the LR+ is not estimable (division by zero) and indicates very strong rule-in value in this cohort. In this context, a κ-FLC index ≥14.6 also showed excellent specificity (96.3%) with a very high LR+ (19.9), indicating strong discriminative capacity for inflammatory intrathecal immunoglobulin synthesis. Reiber’s diagram for k-FLC remained the most sensitive method (91.8%) and showed a specificity of 81.5%, with an LR+ of 4.96. The IgG index showed intermediate accuracy (specificity 85.2%, LR + 4.33).

In the comparison between MS/CIS and the combined non-MS group (OIND + NIND), Reiber’s diagram for k-FLC showed the highest sensitivity (91.8%), with a specificity of 58.7% with an LR+ of 2.22. OCB positivity achieved a sensitivity of 75.5% and a specificity of 84.0%, with an LR+ of 4.72, indicating a more balanced discriminative profile. Similarly, κ-FLC index ≥14.6 showed balanced diagnostic performance (sensitivity 73.5%, specificity 78.7%, LR + 3.40). The lower κ-FLC index cut-off of ≥6.1 remained highly sensitive (85.7%) but showed limited specificity (48.0%), whereas the IgG index again demonstrated lower discriminative ability.

Finally, in the comparison between OIND and NIND, OCB positivity was highly specific (100%) but poorly sensitive (25.0%), yielding an infinite LR +. κ-FLC index ≥14.6 also retained high specificity (96.3%) with modest sensitivity (33.3%) and an LR+ of 8.62. Reiber’s diagram for k-FLC showed a somewhat higher sensitivity (54.2%), although with lower specificity (81.5%) and an LR+ of 2.92. The IgG index showed a similar profile, with a sensitivity of 41.7% and a specificity of 85.2%.

To assess whether blood–CSF barrier function influenced the association betweenintrathecal biomarkers and diagnosis, multivariable logistic regression models were fitted including age, sex, and the κ-FLC index as continuous variables (**Supplementary Table 1**). In the primary model, the κ-FLC index remained independently associated with MS/CIS(OR 1.020 per unit increase; 95% CI 1.010–1.030; p < 0.001). In a sensitivity model additionally adjusting for QAlb rescaled by multiplying by 1000 (QAlb_x1000), the association of the κ-FLC index with MS/CIS remained unchanged (OR 1.020; 95% CI 1.009–1.031; p < 0.001), while QAlb_x1000 was not significantly associated with MS/CIS (OR 1.016; 95% CI 0.925–1.115; p = 0.743). Age and sex were not independently associated with the diagnosis. Detailed results are provided in **Supplementary Table 1**.

## Discussion

In this study, the κ-FLC index proved to be a useful biomarker for differentiating MS/CIS from other inflammatory neurological diseases, with overall diagnostic performance comparable to that reported in previous series (1, 13–16). However, in our study, a higher cut-off value (≈14.6) provided the best balance between sensitivity and specificity for the diagnosis of MS, highlighting the context-dependent nature of optimal threshold selection. Reiber’s diagram for κ-FLC, in turn, showed the highest sensitivity across all comparisons, supporting its value as a complementary method for detecting intrathecal κ-FLC synthesis.

κ-FLCs are produced by B lymphocytes during immunoglobulin synthesis and are physiologically present in excess (approximately 10–40%) relative to heavy chains, being secreted as free molecules. While intrathecal immunoglobulin synthesis is not specific to MS, it fulfills the criterion of dissemination in time within the current diagnostic framework (20) and increases diagnostic certainty when interpreted in the appropriate clinical context. Over the past decade, multiple studies have demonstrated the high diagnostic accuracy of the κ-FLC index for distinguishing MS from other neurological disorders (1, 6, 24–29). Although increased intrathecal κ-FLC synthesis has been described in various inflammatory neurological diseases, patients with MS consistently exhibit higher κ-FLC index values than those with NMOSD, MOGAD, or other demyelinating and inflammatory diseases. While some overlap exists at lower values, markedly elevated κ-FLC index levels appear to be particularly characteristic of MS (2, 13, 16, 30).

This distinction is particularly relevant because OCBs and κ-FLC reflect related but not identical aspects of intrathecal immunoglobulin synthesis. OCBs remain the best-established qualitative marker within the diagnostic framework for MS, whereas κ-FLC provides a quantitative and automatable measure that may facilitate implementation in routine practice (12, 31).

Published studies and the updated 2024 McDonald criteria propose κ-FLC index cut-off values clustering around 6 (approximately 5.8–6.6) (9). However, other reports suggest that higher thresholds (approximately 9–12) may be more appropriate when the comparison group includes inflammatory MS mimics (11, 32, 33). Duranti et al., for example, proposed a cut-off value of 12 to differentiate MS from other inflammatory diseases (33). Dechamps et al. highlighted the overlap of lower κ-FLC index values between MS, NMOSD, and MOGAD, reporting median values of 16.8 in NMOSD and κ-FLC index values >6 in a subset of patients with MOGAD (34). A recent study by Su Hyun Kim et al. (35) proposed a cut-off of 12.5 in an Asian cohort. Taken together, these findings support the concept that optimal κ-FLC index thresholds are influenced by the composition of the comparison group and the underlying epidemiological and clinical context (36).

In our study, a κ-FLC index cut-off value of 6.1 maintained high sensitivity but showed limited specificity for distinguishing MS from other inflammatory neurological diseases. In contrast, a higher threshold of 14.6 achieved a more balanced diagnostic performance, with a sensitivity of approximately 74% and a specificity close to 70%. These findings are consistent with recent reports from other low-prevalence settings, in which upward shifts in optimal κ-FLC index thresholds have been observed to preserve diagnostic specificity when inflammatory MS mimics are common.

The analysis based on the Reiber diagram provides an important methodological perspective. In the primary comparison between MS/CIS and OIND, Reiber’s diagram for κ-FLC increased sensitivity to 91.8%, but specificity decreased to 45.8%, suggesting that this approach may be particularly useful for maximizing sensitivity in the detection of intrathecal κ-FLC synthesis, albeit with a lower capacity to distinguish MS from other inflammatory disorders in this clinically relevant scenario. In this regard, Konen et al. (12) proposed that quotient-diagram approaches may be preferable to a linear κ-FLC index for diagnostic purposes. Nevertheless, because the κ-FLC index is currently incorporated into the McDonald criteria, it remains the method most commonly used in real-world clinical practice. Accordingly, the definition of the most appropriate κ-FLC index cut-off remains an important area of ongoing research, particularly in populations where inflammatory mimics of MS are frequent.

To our knowledge, no studies have been published evaluating this biomarker in Latin American populations. Multiple sclerosis in Latin America presents distinctive epidemiological and clinical characteristics, with a reported incidence ranging from 0.15 to 3 cases per 100,000 person-years and a prevalence of 0.75 to 38.2 cases per 100,000 inhabitants, lower than that reported in Europe and the United States (37–39). In this region, infectious, metabolic, and autoimmune conditions that can mimic MS, such as syphilis, HIV infection, neurotuberculosis, neurocysticercosis, HTLV-1 infection, arboviral diseases, vitamin B12 and copper deficiencies, and NMOSD, are relatively more frequent and must be carefully excluded during the diagnostic process (40, 41).

In addition, we explored whether blood–CSF barrier function could influence the association between intrathecal biomarkers and diagnosis by incorporating QAlb into multivariable models. In logistic regression analyses adjusted for age and sex, the κ-FLC index remained independently associated with MS diagnosis, whereas QAlb showed no significant association. This finding suggests that the diagnostic value of the κ-FLC index in our study is not primarily driven by passive diffusion across a disrupted blood–CSF barrier, but rather reflects true intrathecal B-cell activity. These results are in line with previous studies indicating that κ-FLC index outperforms both QAlb-adjusted measures and serum-based indices for identifying intrathecal immunoglobulin synthesis in MS. Prior work has shown that elevated QAlb may be common in infectious or systemic inflammatory conditions affecting the central nervous system, potentially confounding the interpretation of CSF biomarkers when barrier dysfunction predominates (42–44). Our findings support the use of the κ-FLC index as a robust marker that retains diagnostic relevance even after accounting for blood–CSF barrier integrity, reinforcing its clinical utility in heterogeneous inflammatory populations. Beyond inflammatory demyelinating disorders, emerging evidence suggests that κ-FLC measurements may also have broader applicability in other neuroinflammatory settings, including selected psychiatric syndromes associated with autoimmune mechanisms (45, 46).

Evaluating the performance of the κ-FLC index in local patient populations, within their specific clinical and healthcare contexts, is therefore essential before its widespread clinical implementation. An additional advantage of the κ-FLC index is its quantitative and automatable nature. Measurement is rapid, cost-effective, and independent of operator interpretation (47, 48), which is particularly relevant in low- and middle-income countries where access to specialized laboratory techniques may be limited.

The IgG index showed substantially lower diagnostic performance, as also reported by other groups (23), reinforcing current recommendations to prioritize κ-FLC index and CSF oligoclonal bands in the initial diagnostic algorithm.

This study has limitations. A larger sample size would allow for more precise estimates of diagnostic metrics and narrower confidence intervals. Furthermore, as this is a single-center study, the reproducibility of the proposed thresholds should be validated in larger and more diverse populations.

One of the strengths of this study is that it provides real-world evidence on the performance of the κ-FLC index as an intrathecal biomarker in a Latin American series, describing a cut-off value that differs from those commonly reported in European and North American populations. Our findings demonstrate that the κ-FLC index is a useful biomarker with diagnostic performance comparable to established CSF biomarkers, representing a valuable complementary tool in our region.

These unresolved methodological and clinical questions are currently being addressed in an ongoing prospective multicentric real-world study (NCT07183020), specifically designed to compare the diagnostic sensitivity of CSF κ-FLC and OCBs and to assess whether Reiber’s diagram is superior to the κ-FLC index threshold of 6.1 for detecting intrathecal synthesis (49). The results of this initiative may be especially relevant for interpreting single-center studies such as ours and for defining more robust, internationally applicable recommendations regarding the complementary use of OCBs, κ-FLC indices, and quotient-diagram approaches.

## Data Availability

The original contributions presented in the study are included in the article/**Supplementary Material**. Further inquiries can be directed to the corresponding author.

## Supplementary material

Supplementary Table 1 Binary logistic regression models for MS/CIS. Model 1 (primary): adjusted for age and sex. Model 2 (sensitivity): adjusted for age, sex, and rescaled QAlb (×1000). *Outcome: MS/CIS = 1; OIND+NIND = 0. Sex coded as 1 = female, 2 = male (therefore the OR for sex corresponds to the change from female to male). OR, odds ratio; CI, confidence interval; SE, standard error; QAlb, CSF/serum albumin quotient. *QAlb was rescaled by multiplying by 1000 (QAlb_x1000) to improve numerical stability and interpretability of the odds ratios.*
