# Supplementary material for: Kappa free light chain index in multiple sclerosis and other inflammatory CNS diseases: a pilot single-center study from Argentina
Source: Front Immunol. 2026 May 29;17:1803446. doi: 10.3389/fimmu.2026.1803446 (PMC13259835; doi:10.3389/fimmu.2026.1803446)
Supplement: Supplementary Table 1 — Binary logistic regression models for MS/CIS. Model 1 (primary): adjusted for age and sex. Model 2 (sensitivity): adjusted for age, sex, and rescaled QAlb (×1000). Outcome: MS/CIS = 1; OIND+NIND = 0. Sex coded as 1 = female, 2 = male (therefore the OR for sex corresponds to the change from female to male). OR, odds ratio; CI, confidence interval; SE, standard error; QAlb, CSF/serum albumin quotient. *QAlb was rescaled by multiplying by 1000 (QAlb_x1000) to improve numerical stability and interpretability of the odds ratios. [file Table1.docx]

Supplementary Table 1. Binary logistic regression models for MS/CIS

Model 1 (primary): adjusted for age and sex

| **Variable** | **B** | **SE** | **Wald** | **df** | **p value** | **OR (Exp(B))** | **95% CI for OR** |
| --- | --- | --- | --- | --- | --- | --- | --- |
| Age | −0.023 | 0.014 | 2.829 | 1 | 0.093 | 0.977 | 0.952–1.004 |
| Sex | 0.442 | 0.464 | 0.907 | 1 | 0.341 | 1.556 | 0.626–3.863 |
| κ-FLC index | 0.020 | 0.005 | 14.753 | 1 | <0.001 | 1.020 | 1.010–1.030 |
| Constant | −0.925 | 0.915 | 1.022 | 1 | 0.312 | 0.396 | — |

Model 2 (sensitivity): adjusted for age, sex, and rescaled QAlb (×1000)

| **Variable** | **B** | **SE** | **Wald** | **df** | **p value** | **OR (Exp(B))** | **95% CI for OR** |
| --- | --- | --- | --- | --- | --- | --- | --- |
| Age | −0.019 | 0.014 | 1.821 | 1 | 0.177 | 0.981 | 0.954–1.009 |
| Sex | 0.549 | 0.478 | 1.321 | 1 | 0.250 | 1.732 | 0.679–4.422 |
| κ-FLC index | 0.020 | 0.005 | 13.925 | 1 | <0.001 | 1.020 | 1.009–1.031 |
| QAlb_x1000* | 0.016 | 0.047 | 0.107 | 1 | 0.743 | 1.016 | 0.925–1.115 |
| Constant | −1.272 | 1.006 | 1.599 | 1 | 0.206 | 0.280 | — |

*Outcome: MS/CIS = 1; OIND+NIND = 0. Sex coded as 1 = female, 2 = male (therefore the OR for sex corresponds to the change from female to male). OR, odds ratio; CI, confidence interval; SE, standard error; QAlb, CSF/serum albumin quotient.*

**QAlb was rescaled by multiplying by 1000 (QAlb_x1000) to improve numerical stability and interpretability of the odds ratios.*

Figure 3. ROC curve of the different diagnostic methods.
